# A radiocarbon spike at 14 300 cal yr BP in subfossil trees provides the impulse response function of the global carbon cycle during the Late Glacial

**DOI:** 10.1098/rsta.2022.0206

**Published:** 2023-11-27

**Authors:** Edouard Bard, Cécile Miramont, Manuela Capano, Frédéric Guibal, Christian Marschal, Frauke Rostek, Thibaut Tuna, Yoann Fagault, Timothy J. Heaton

**Affiliations:** ^1^ CEREGE, Aix-Marseille University, CNRS, IRD, INRAE, Collège de France, Technopôle de l'Arbois, BP 80, 13545 Aix-en-Provence, France; ^2^ IMBE, Aix-Marseille University, CNRS, IRD, Avignon University, Technopôle de l'Arbois, 13545 Aix-en-Provence, France; ^3^ Department of Statistics, School of Mathematics, University of Leeds, Leeds LS2 9JT, UK

**Keywords:** radiocarbon, dendrochronology, carbon cycle, solar variations, solar energetic particles, Miyake event

## Abstract

We present new ^14^C results measured on subfossil Scots Pines recovered in the eroded banks of the Drouzet watercourse in the Southern French Alps. About 400 new ^14^C ages have been analysed on 15 trees sampled at annual resolution. The resulting Δ^14^C record exhibits an abrupt spike occurring in a single year at 14 300–14 299 cal yr BP and a century-long event between 14 and 13.9 cal kyr BP. In order to identify the causes of these events, we compare the Drouzet Δ^14^C record with simulations of Δ^14^C based on the ^10^Be record in Greenland ice used as an input of a carbon cycle model. The correspondence with ^10^Be anomalies allows us to propose the 14.3 cal kyr BP event as a solar energetic particle event. By contrast, the 14 cal kyr BP event lasted about a century and is most probably a common Maunder-type solar minimum linked to the modulation of galactic cosmic particles by the heliomagnetic field. We also discuss and speculate about the synchroneity and the possible causes of the 14 cal kyr BP event with the brief cold phase called Older Dryas, which separates the Bølling and Allerød millennium-long warm phases of the Late Glacial period.

This article is part of the Theo Murphy meeting issue 'Radiocarbon in the Anthropocene'.

## 1. Introduction

The radiocarbon method is widely used to date fossil samples over the last 55 000 years. It is based on the radioactive decay of ^14^C produced in the upper atmosphere by interaction with cosmic ray particles. The ^14^C/^12^C ratio of the fossil sample is compared with the atmospheric ^14^C/^12^C ratio, which is the starting point from which ^14^C decays exponentially through radioactive decay with a half-life of 5700 years. However, radiocarbon dating is not exact because the atmospheric ^14^C/^12^C ratio has varied through time owing to changes in the rate of production by cosmic rays, as well as rearrangements of the biogeochemical carbon cycle.

To accurately calculate the calendar age of the fossil sample, it is necessary to know the ^14^C/^12^C ratio of the contemporary atmosphere at the sample's time of growth. The raw radiocarbon age is therefore corrected (i.e. calibrated) by comparing the measured ^14^C content in the fossil sample of unknown age with those of other samples for which accurate and precise ages have been measured by independent methods such as counting the annual rings of trees, annual laminations in varved sediments or absolute dating by the uranium–thorium method. For 30 years, radiocarbon calibration curves have been prepared by the international working group IntCal, and the latest iteration (IntCal20) was published in 2020 [1,2].

Many records from various archives are combined to construct the ^14^C calibration curves, but the most precise and accurate are based on dendrochronologically dated tree-ring series. Subfossil trees are abundant for the Holocene starting at ≈11 600 yr before present (BP, present = 1950 common era [CE], [3]). These have mainly been discovered in peat bogs and dredged out of gravel pits in the alluvial plains of the large rivers of central Europe (Danube, Main, Rhine, [4,5]). Through matching tree ring width patterns of numerous overlapping sections, they have allowed for the construction of a continuous calibration curve, absolutely dated with annual precision, going back to 12 325 cal yr BP [6]. For times before the Holocene, the availability of trees is limited, in particular for the cold episodes of the Late Glacial during which the forests regressed (e.g. the Younger Dryas). Dendrochronological Late Glacial sequences have come mainly from Switzerland and France [7–9]. To date, the cold climatic context of the Pleistocene has made discoveries of subfossil trees extremely rare, except in the Southern Hemisphere [10]. The oldest tree-ring series are known as *floating* [11] since, while their constituent rings can be counted to create a relative internal chronology, they cannot be dendro-matched with the main Holocene absolute chronology. However, ^14^C analyses performed at high resolution on overlapped absolute and floating tree-rings series enable one to link them almost absolutely and hence to extend the calibration on annual tree rings until ≈13 900 cal yr BP [1,10,12].

The first source of complexity when converting ^14^C determinations to calendar ages is the variable production of the ^14^C isotope. Most ^14^C production occurs in the lower stratosphere and upper troposphere with nuclear (n-p) reactions between cosmic ray neutrons and atmospheric nitrogen. These secondary neutrons are formed originally by the collision between atmospheric gases and the protons that constitute a significant portion of the primary cosmic radiation originating from our galaxy. The arrival on Earth of these charged particles is modulated at centennial to decadal scales by variations of magnetic properties of the solar wind, leading to lower cosmogenic production during high solar activity phases, and making atmospheric ^14^C a mirror image of solar activity. These changes are superimposed on slow variations due to geomagnetic field changes.

This production variability is further modified and distorted by mixing of ^14^CO_2_ molecules in the global carbon cycle. For example, while the 11-yr sunspot cycle is accompanied by a strong variation in ^14^C production (≈ ± 200‰), the ^14^C/^12^C cycle measured in the atmosphere (and tree-rings) remains small (±2‰) due to the damping effect by the global carbon cycle [13,14]. Changes in ocean circulation could also have affected the outgassing of old, ^14^C-depleted CO_2_.

In addition to the heliomagnetic and geomagnetic modulations of galactic cosmic rays, it has been proposed recently that abrupt ^14^C production maxima could be linked to short-term energetic particle bursts released by solar flares and coronal mass ejections from the Sun. The first solar energetic particle (SEP) spike identified was for the year 774 CE and manifested as an abrupt ^14^C step (15‰ ≈ 120 ^14^C yr) occurring over a single year in recent trees from Japan [15]. The existence of the 774 CE event at global scale has since been confirmed by measurements on many other trees from different locations spread over the planet. In addition, corresponding annual spikes for other cosmogenic isotopes (^10^Be and ^36^Cl) have been found in polar ice cores from Greenland and Antarctica [16], providing further information to characterize the particle flux.

The discovery of the 774 CE spike fostered new ^14^C measurement programmes on tree ring series from the Holocene at annual resolution. This is an enormous task since, before this discovery, tree-ring calibration had mainly been based on ^14^C ages measured on decadal wood sections. So far, four SEP spikes have been evidenced with multiple cosmogenic isotopes, 774 CE (1176 Cal BP), 993 CE (957 Cal BP), 2610 Cal BP, 9125 Cal BP) and a few others have been proposed based solely on ^14^C, 1052 CE (898 Cal BP), 1279 CE (672 Cal BP), 7210 Cal BP, 7360 Cal BP [17].

As a follow-up of our previous work to extend ^14^C calibration during the Younger Dryas period based on subfossil pines from the French Southern Alps [18,19], we have measured ^14^C in trees at annual resolution over a 700-yr window belonging to the Bølling-Allerød period. The new ^14^C record can be compared with ^10^Be data from Greenland ice in order to identify variations linked to cosmogenic production changes, notably at *ca* 14.3 cal kyr BP during an abrupt event proposed as a new SEP spike. This comparison is also useful to evaluate the influence of the carbon cycle in the heart of the deglacial period. Indeed, the ^14^C signal due to an abrupt production spike constitutes the impulse response function characterizing the entire global carbon cycle (e.g. [20]).

## 2. Sites and materials

**Figure 1.**
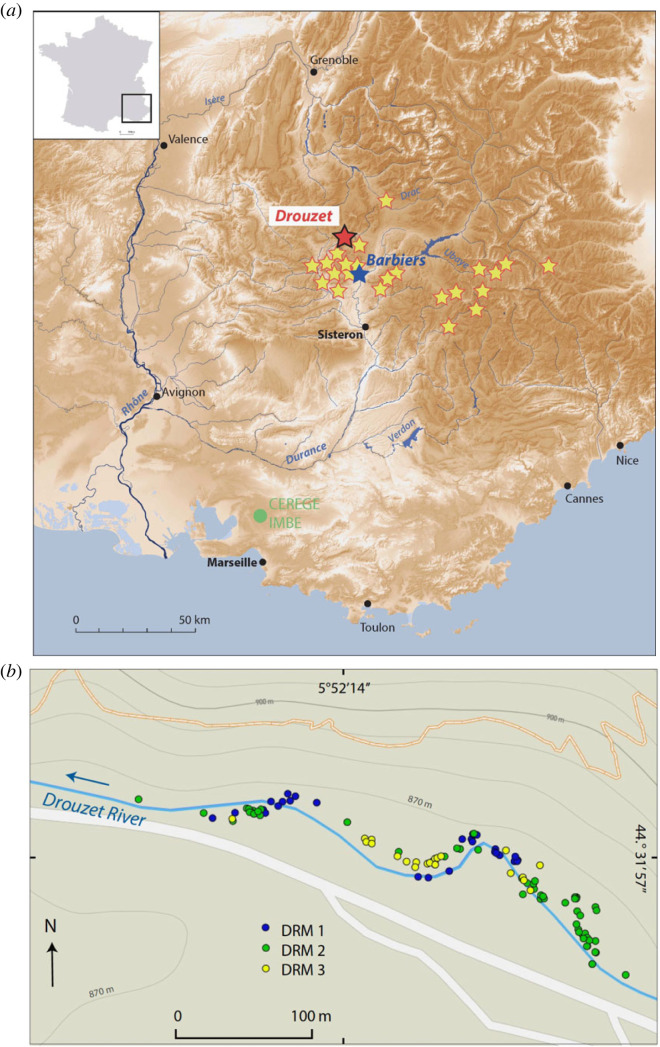
(*a*) Map of Southeastern France showing the locations of the subfossil tree deposits in the middle Durance region, including Barbiers and Drouzet rivers. (*b*) Close up on Drouzet River (GPS coordinates and tree labels are available in the electronic supplementary material, table S2).

**Figure 2.**
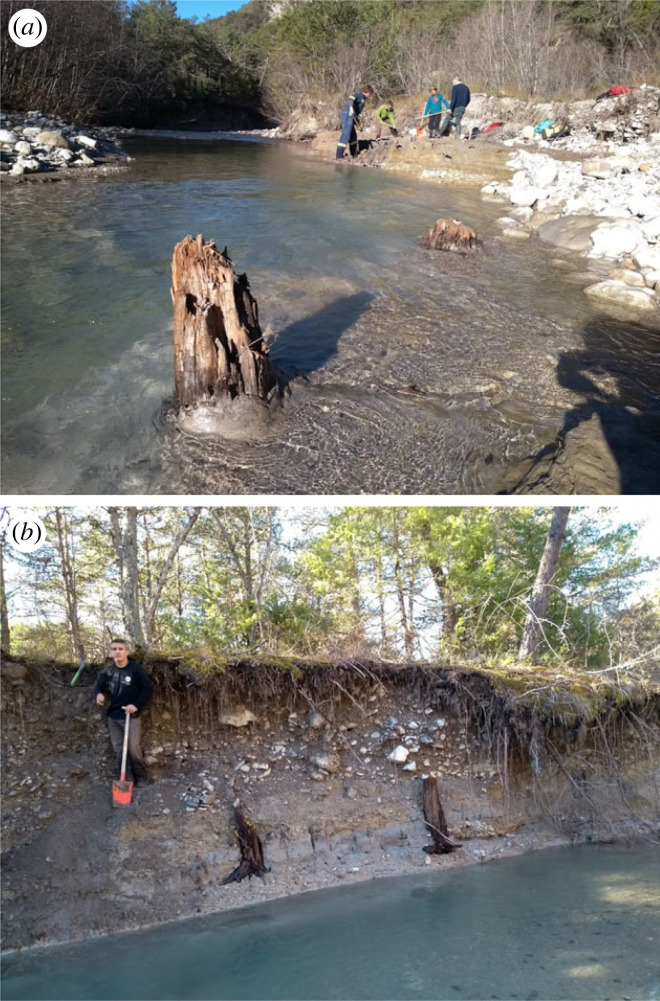
(*a* and *b*) Photographs of the Drouzet river showing subfossil trees (Scots Pines) buried in alluvial deposits. Trees appear rooted at different levels in the main stream or in the river banks.

Over the past 25 years, we conducted intensive fieldwork to collect subfossil trunks in order to complement the wood collection stored at IMBE in Aix-en-Provence ([7] and follow-up papers, figure 1*a*). In the region of the upper course of the Durance River in the Southern French Alps, many subfossil Scots Pines (*Pinus sylvestris* L.) were discovered in alluvial sediments dated from Late Glacial to the early Holocene (figure 2). Their noteworthy fossilization and preservation can be explained by a combination of geologic, topographic and climatic factors: (i) bedrock consists of highly erodible calcareous marl; (ii) steep slopes are widely developed in this alpine area; and (iii) this region is subject to stormy rainfall influenced by both Mediterranean and mountain climates.

After the Late Glacial Maximum, dated in the area at ≈21 cal kyr BP [21], rapid warming accompanied by increased precipitation led to an intense erosion of slopes, generating colluvial to alluvial sedimentation and the formation of wide and deep alluvial fans characteristic of the sub-Alpine landscapes. This created the so-called ‘Main Postglacial Infilling' (MPI), which took place between 14.5 and 7 cal kyr BP [22–24]. Throughout the MPI, with its high sedimentation rate, many trees were buried (mainly *Pinus sylvestris* L., which represents Late Glacial and early Holocene pioneer vegetation). These trees stayed well preserved and buried until their recent exposure following the vertical incision of the rivers. Erosion of river banks during the winter season reveals new subfossil trees almost every year.

In total, 172 subfossil trees were discovered in a 500 m-long and 30 m-wide stretch of the Drouzet River (44°31'57^″^ N 5°52'14^″^ E) (figure 1*b*). Trees are buried in loamy deposits of the MPI forming a 2 m thick alluvial terrace. This terrace contains three rooting levels that are very difficult to differentiate because the loamy alluvial deposits are lenticular shaped, discontinuous and partially preserved. In addition, the riverbed incision in the Drouzet river is not uniform. Consequently, some trees have exposed roots whereas in other cases, only the upper part of the trunk protrudes from the water (figure 2). All trees are Scots Pines (*Pinus sylvestris* L.) and they still stand rooted *in situ*, except two trees that had been carried away downstream. Most of the trees still have pieces of bark remaining. The height of the remaining trunks ranges from a few centimetres to 1 m high. Their diameters range between 59 and 5 cm with an average of 27 cm.

Each subfossil trunk has been extracted from the sediment with a pickaxe and described (height, circumference, state of preservation, GPS coordinates (electronic supplementary material, table S1)). Whenever possible, two or three discs 5–10 cm thick were sampled with a chainsaw, one just above the root to estimate the germination date, and one higher on the stem to measure ring-widths while avoiding the distortions near the collar.

## 3. Dendrochronological analyses

Out of Drouzet trunks, 140 trees were sampled for dendro analysis while 32 poorly preserved trees were discarded. When the wood was hard and well preserved, discs were air-dried and sanded by using progressively finer sandpaper up to 400 grain size. Damaged and waterlogged wood samples were wrapped in plastic sheeting and scraped with a razor blade to obtain plane surfaces and make rings visible. Standard dendrochronological techniques were employed in chronology development. Ring-widths (TRW) were measured on at least three radii using a LINTAB measuring system with a precision of 0.01 mm and the TSAPWin software [25]. All chronologies were built by using standard statistical tests and visual comparison of the raw TRW curves: the percentage of parallel variation or *Gleichläufigkeit* (Glk) [26] and the *t* values obtained with Baillie and Pilcher indices [27] and Hollstein indices [28] (electronic supplementary material, table S2).

The average age of the trees, including pith and bark, is 161 (max 288, min 60) yrs. Generally young trees formed very narrow rings which could mean they recruited on fresh alluvial deposits without organic matter and perhaps within a high-density stand. The last 20–80 years are frequently characterized by very narrow rings, which shows that pines grew slowly due to asphyxia by burial in alluvium and water. Moreover, tree growth is disturbed by hydrological changes and geomorphological stress due to frequent river floods. Therefore, matching of ring patterns was often challenging, even between trees that grew next to each other. Missing rings are frequent in *Pinus sylvestris* species, even more so in such subfossil pines which suffered from recurrent stress. Considering those difficulties, cross-dating was done following two steps: first, between nearby trees and afterwards with more distant trees.

**Figure 3.**
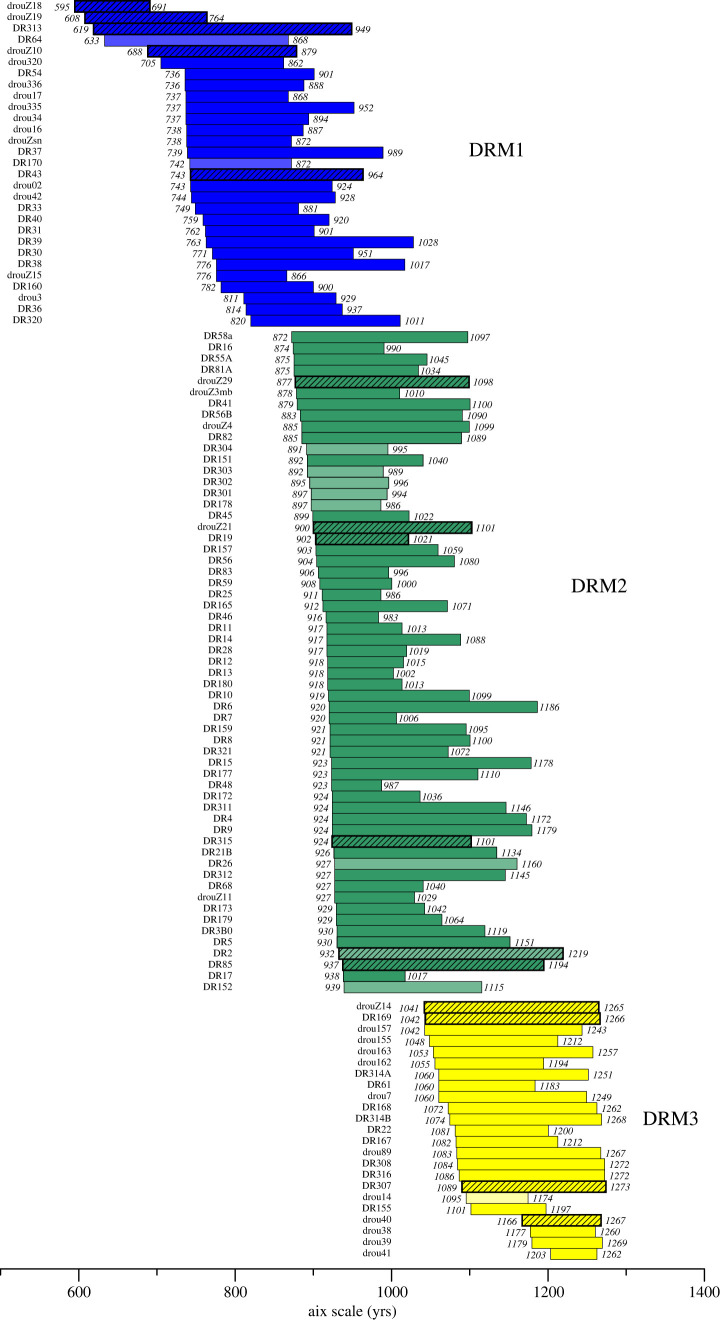
Bar diagram of trees included in the Drouzet chronology reported on the relative Aix scale. The bar diagram reports all cross-dated trees. Lighter bar colour shows correct but weaker matches. The hatched bars refer to trees that were ^14^C dated. The colours (blue, green, yellow) correspond to the three groups of trees that are reliably cross-dated by dendrochronological analysis (correlation parameters for cross-dated Drouzet trees are available in the electronic supplementary material, table S3). Attempts to cross-date DRM1, DRM2 and DRM3 mean chronologies were still tentative.

The dendrochronological analyses made it possible to gather 111 trees from Drouzet into three floating chronologies covering about 680 yrs: DRM1, DRM2 and DRM3 with durations of 434, 348 and 233 years, each including 29, 59 and 23 individual trees, respectively (figure 3). Twenty-nine trees could not be cross-dated, either because ring-width series were too short (40–80 yrs), or because irregular growth prevented a reliable cross-matching.

DRM1 is the oldest group of trees. They are located mostly downstream in the river or in the middle part of the studied area (figure 1*b*). DRM1 trees belong to the lower stratigraphic level and their roots are still buried deep in the late glacial deposit. The mean length of ring-width series is 173 (min 91, max 331) yrs. Two subgroups of trees appear, from drouZ18 to DR64 and from drouZ10 to DR320, attesting to two phases of germination. Statistical test values between trees of the oldest subgroup are 58 < Glk < 63, 2.2 < *t*BP < 5.4 and 2.5 < *t*H < 4.1, except for DR64, which better matches with the youngest subgroup. In this last subgroup, most of the trees cross-date with statistical values of Glk > 60, *t*BP and *t*H > 4, except for tree DR170, which shows a weaker correlation (electronic supplementary material, table S2).

DRM2 consists of 59 trees that colonized the alluvial plain over a period of 70 years. Most of them are located upstream in the eastern part of the site. The mean length of ring-width series is 159 (min 65, max 288) yrs. Trees cross-match well (Glk up to 7 and *t*BP - *t*H > 5.5 for the best correlations). Statistical test values are weaker for trees DR152, DR26, DR2 and few short chronologies.

DRM3 is the youngest group of trees located in the upper stratigraphic level, which is in the middle part of the site. With a mean value of 155 (min 60, max 255) rings, DRM3 is composed of trees younger than those of DRM1 and DRM2. Young trees continuously recruited over a period of 60 years. They are followed by a group of four trees whose position is confirmed by radiocarbon.

Attempts to cross-match the three floating chronologies DRM1, DRM2 and DRM3 were still tentative. It is supposed that the DRM1, DRM2 and DRM3 groups reflect three main germination phases following the deposit of alluvial sediments in the river. This explains why the correlation between ring-width series belonging to different groups is so difficult: comparing old dying trees with young trees in their juvenile phase is very challenging. Although the chronologies may be synchronous, it is difficult to evidence by means of tree-ring patterns. Nevertheless, tree DR320 could theoretically link DRM1 to DRM2, as (i) it cross-dates with the trees DR46, DR180 and DR17 (60 < GLk < 68, 4 < *t*BP < 4.8 and 3.5 < *t*H < 4.6); and (ii) trees drou3 and DR38 from DRM1 cross-date with DR19, DR58a from DRM2 (Glk > 60 and *t* > 3.2). Besides that, the link between DRM2 and DRM3 is weaker, even if trees DR26 and DR312 (from DRM2) cross-date with two trees from DRM3. Although statistical agreements between DRM1, DRM2 and DRM3 are not sufficiently high, visual agreements between their ring-width curves are considered reliable enough to provide a first guess for their relative position. These initial positions of the three chronologies are strengthened and refined by using their ^14^C measurements as detailed below.

## 4. Radiocarbon methods

In selected trees, the wood from each annual ring was separated using a scalpel under a binocular microscope and sliced into small pieces. About 100 mg of dry wood for each sample was pretreated for ^14^C analysis using methods previously developed for trees from the nearby site of Barbiers, which covers the Allerød-YD transition [18,19] and was included in the IntCal20 calibration curve [1].

After reviewing the literature on wood pretreatments and performing numerous tests on modern and subfossil wood from coniferous and broadleaf trees [18], we selected a procedure of holocellulose extraction with acid-base-acid-bleaching pretreatment (ABA-B). This method consists of a classic ABA treatment, with solutions of HCl and NaOH at 4% concentration, followed by a bleaching step performed with 60 g of NaClO_2_ in 1 l of ultrapure water in acid solution (HCl) at pH 3. The choice of this protocol was based on its limited duration and complexity, and its excellent and reproducible analytical results as shown by ^14^C results, ^13^C /^12^C ratios, carbon % and overall mass yield % [18].

After chemical pretreatment, the dried residues of wood were weighed in tin capsules, then combusted with an elemental analyser, and the evolved CO_2_ was finally transformed into graphite with an AGE3 system. The graphite target was then analysed for its ^14^C/^12^C and ^13^C/^12^C ratios using the AixMICADAS system [29]. Standards (OxA2 NIST SRM4990C) and blanks (VIRI-K, pretreated like the other wood samples) were processed together with samples and used for normalization and blank correction, respectively. In addition, IAEA-C3 (cellulose pretreated like the wood samples) and IAEA-C8 (oxalic acid) standards were measured for ^14^C in the same batches, serving as control standards.

High precision ^14^C measurements were performed with AMS runs ≈ 50% longer than usual in order to reach at least 800 000 ion counts for each OxA2 standard target on the same magazine. An additional uncertainty of 1.6‰ was propagated in the ^14^C analytical errors and background correction following the convention described in Capano *et al*. [18]. The data are reported in terms of conventional ^14^C age in years BP [30].

As an initial control on data quality, we dated the wood samples of the Sixth International Radiocarbon Intercomparison (SIRI, see [18] for the results compared with consensus values). In addition, we participated in an international intercomparison on single-year tree-ring series [31], which aimed to investigate possible offsets between AMS laboratories at high precision. The results show that the CEREGE radiocarbon unit produces precise results that are consistent with their stated uncertainties.

## 5. Radiocarbon results

Radiocarbon ages were measured with AixMICADAS on *ca* 400 samples from 15 trees selected from the three DRM1, DRM2 and DRM3 series (table S1 in the electronic supplementary material for numerical results). In general, we dated samples from every third annual ring, although higher resolution (dating every consecutive annual ring) was used to study specific details. Several Drouzet trees exhibit very narrow rings, forcing us to mix two or three annual rings to produce reliable graphite targets. In addition, the outer wood of several trees was clearly altered as testified by their very low mass yield during chemical pretreatment, preventing the synthesis of reliable targets or correct measurements by AMS.

**Figure 4.**
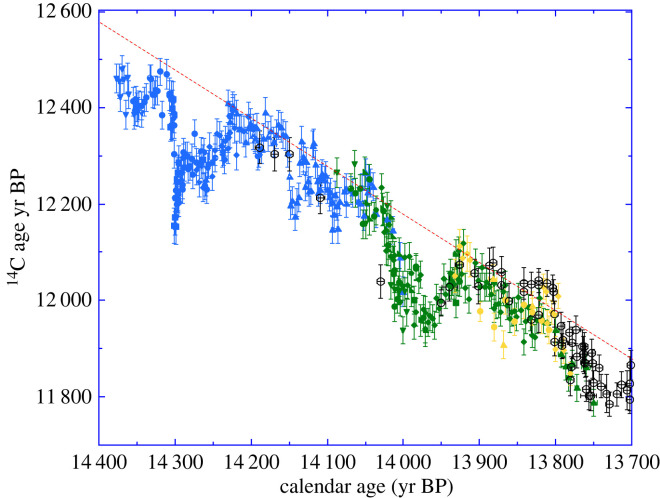
Radiocarbon ages of Drouzet tree samples of the DRM1 (blue), DRM2 (green) and DRM3 (yellow) sequences placed on the preliminary chronology shown in figure 3 visually correlated to the German pine absolutely dated chronology shown with black open dots [1,32]. The (*y*-axis) error bars for ^14^C ages are shown at 1–σ. The number of annual rings measured in each sample is represented by the (*x*-axis) bars (e.g. ±0.5 yr for annual ring samples for most Drouzet analyses, ±5 yr for decadal measurements). The red dashed line shows the 1 : 1 slope of the ^14^C-Cal age relationship.

Figure 4 shows the results spanning the time window 12.6–11.7 ^14^C kyr BP corresponding to 14.4–13.75 cal kyr BP based on a preliminary visual matching with the Late Glacial German pine chronology also shown in figure 4 [1,32]. The new data analysed on annual samples agree with eight previous ^14^C ages on decadal samples of Drouzet wood measured by beta-counting in Heidelberg [8]. The overlap between the ^14^C results on DRM3 and DRM2 (yellow and green points) covers more than 150 cal yrs. The overlap between DRM2 and DRM1 (green and blue points) is shorter (≈100 cal yrs) but it includes a common sharp drop of ^14^C ages around 14.0 cal kyr BP.

Overall, the Drouzet record shows the expected long-term trend linked to the ^14^C radioactive decay on which are superimposed brief anomalies towards younger ^14^C ages around 14.35, between 14.3 and 14.2, around 14.13 and between 14.0 and 13.9 cal kyr BP. A prominent feature of the record is an abrupt drop of ^14^C ages at 14.3 cal kyr BP.

To provide a more precise and refined matching of the three floating DRM ^14^C sequences, both to one another and to the absolutely dated (known-age) Late Glacial German pine chronology [1,32] we implemented a Bayesian Markov Chain Monte Carlo (MCMC) scheme. This method was based upon that used to place the floating tree ring sequences within the IntCal20 calibration curve [12]. The three floating DRM ^14^C sequences were combined with the absolutely dated German ^14^C chronology. The starting calendar ages of each of the three floating DRM sequences (denoted *T*_1_, *T*_2_ and *T*_3_) were considered unknown, along with the true value of the atmospheric Δ^14^C curve from 14 400 to 12 750 cal yr BP (which was modelled by a Bayesian cubic spline, placing knots every 3 years, with coefficients β and smoothing parameter *λ*). The method then aimed to find plausible placements for the DRM ^14^C sequences that both matched with one another and with the known-age German pine ^14^C measurements, on the basis all the measurements must share a common Δ^14^C atmosphere. Using all the ^14^C data within the same MCMC fitting scheme is key as, due to the overlap between all ^14^C sequences, the placement of each DRM sequence informs on the location of the others.

**Figure 5.**
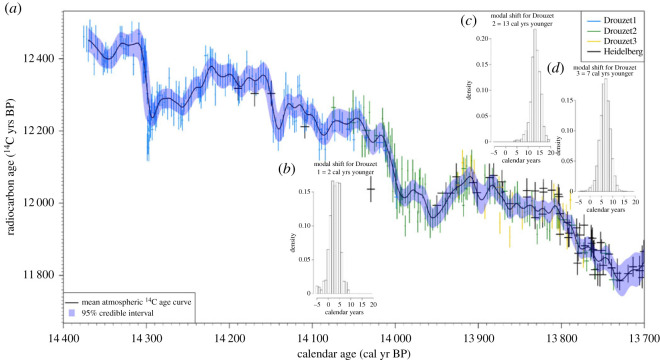
(*a*) Radiocarbon ages of Drouzet tree samples of the DRM1 (blue), DRM2 (green) and DRM3 (yellow) sequences placed on the new chronology with our Bayesian MCMC scheme (described here and [12]) for matching to the German pine absolutely dated chronology shown in black [1,32]. The DRM1, DRM2 and DRM3 sequences are plotted at their modal (most likely) calendar age locations. The (*y*-axis) error bars for ^14^C ages are shown at 1-σ. The number of annual rings measured in each sample is represented by the (*x*-axis) bars (e.g. ±0.5 yr for annual ring samples for most Drouzet analyses, ±5 yr for decadal measurements). The black curve with 95% credible interval (shaded blue envelope) shows the posterior mean estimate for the atmospheric ^14^C age based on the Drouzet and German pines obtained within the MCMC scheme. This curve is modelled as a cubic spline and is attenuated since it averages over all potential calendar age fitting locations for the DRM sequences. (*b*–*d*) The three inserts show the posterior calendar age histograms for the DRM1, DRM2 and DRM3 sequences relative to their preliminary placements based on tree ring patterns.

Metropolis-within-Gibbs was used to alternate between updating the starting ages of DRM sequences {*T*_1_, *T*_2_, *T*_3_}, the spline β and the smoothing parameter *λ*. The method was adapted to incorporate the varying level of blocking within the ^14^C measurements exactly (i.e. that some measurements represented the average of multiple annual growth rings, while others were single year). The fit of the ^14^C observations to the unknown, common, atmosphere was assessed in the symmetric *F*^14^C domain. Uninformative priors were placed on each DRM age *T_i_* and the smoothing parameter *λ*. The method was then run for 100 000 iterations with the first 50 000 discarded as burn-in. The results are shown in figure 5. In panels *b*, *c* and *d*, we present the posterior estimates for the calendar age shifts from the preliminary DRM placements based on visual matching, while the corresponding posterior mean estimate of cal age versus ^14^C age obtained by our MCMC scheme during the above fitting can be seen in the main panel *a*. The posterior estimate for the shift to the calendar age of DRM1, compared with its initial visual placement, indicate it should be moved 2.9 ± 2.4 (mean ± 1*σ*) cal yrs younger; for DRM2 the suggested shift is for the sequence to be moved 13.2 ± 2.4 cal yrs younger; and for DRM3 it is 6.6 ± 2.3 cal yrs younger. The most probable (the mode of the posterior marginal) calendar age shift for each Drouzet sequences, optimizing agreement with each other and the known-age German ^14^C chronology, would move DRM1 younger by 2 cal yrs, DRM2 younger by 13 cal yrs and DRM3 younger by 7 cal yrs from their initial placements based on visual matching.

A simplified placement was also performed, assuming that the relative positions of DRM1, DRM2 and DRM3 within the preliminary placement is accurate (i.e. that DRM1, DRM2 and DRM3 form a single chronology based on independent dendrochronological matching). Our MCMC scheme of matching to the German absolute chronology suggested a shift of this single DRM sequence, from its preliminary placement, by 4 ± 1.5 cal yrs (with a mode of 5 cal yrs) towards a younger age.

The posterior mean curve of figure 5*a* (and its accompanying credible intervals) averages over all possible placements of the floating DRM data. Due to this placement averaging, the mean curve attenuates the variability in the atmospheric Δ^14^C from one year to the next. This can most clearly be seen in the substantial reduction in amplitude when reproducing the sharp change in the ^14^C of DRM1 around 14.3 cal kyr BP. The curve shown in figure 5*a* is further attenuated since the Δ^14^C is modelled by a standard cubic spline. Such splines are not well-designed to represent extremely sharp discontinuities. This should not however impact upon the MCMC placing of the DRM data since the sharp change only affects the oldest part of DRM1 and not the other DRM sequences. To remove the confounding effect of this placement-averaging on the posterior mean of the atmospheric curve, we must consider a fixed calendar age placement of theDRM data.

**Figure 6.**
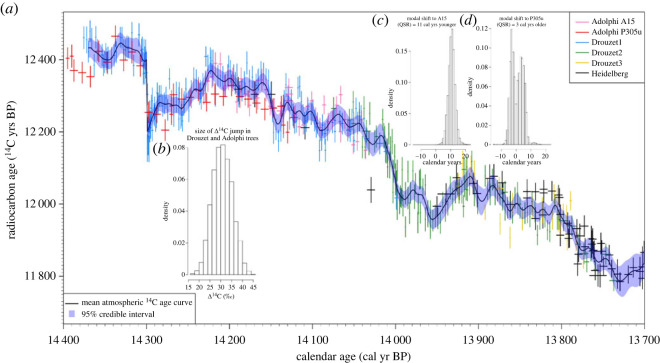
(*a*) Radiocarbon ages of P305u (red) and A15 (pink) Italian trees placed on the new chronology with our MCMC scheme for matching to the DRM1 (blue), DRM2 (green) and DRM3 (yellow) sequences set at their, optimal, posterior mode calendar ages of figure 5. Error bars are shown at 1–σ for ^14^C ages. The number of annual rings measured in each sample is represented by the (*x*-axis) bars (e.g. ±0.5 yr for annual ring samples for most Drouzet analyses, ±5 yr for decadal measurements). The black curve with 95% credible interval (shaded blue envelope) shows the posterior mean estimate for the atmospheric ^14^C age based on Drouzet, A15 and P305u and German pines using the MCMC scheme. The curve is modelled by a cubic spline to which a Δ^14^C step function has been added between 14 300 and 14 299 cal yr BP to investigate the sharp jump in observed ^14^C (which occurs over the course of this year if DRM1 is placed at its most likely, modal, calendar age). The posterior for the size of this jump is shown in insert (*b*)*,* while (*c*) and (*d*) show the two posterior calendar age histograms for A15 and P305u sequences relative to their placements by Adolphi *et al*. [33].

If we place each DRM sequence at its most likely (modal) calendar ages (obtained by the matching to Heidelberg described above), DRM1 indicates the possibility of an abrupt annual change in atmospheric Δ^14^C occurring between the years 14 300 and 14 299 cal yr BP. To assess the level of evidence for such a sharp production change, we modify our base statistical model for Δ^14^C. We retain the underlying, smooth, cubic spline component but add a step function (of unknown size *γ*) at this fixed calendar date. Another MCMC scheme (similar to that implemented above) is then fitted—treating the spline coefficients β and smoothing parameter *λ* as unknown, as well as the size of the step function *γ* in Δ^14^C units. The results are seen in figure 6*a* with its insert 6*b* showing our posterior estimate for the step amplitude *γ*.

When estimating this (modal-age) curve, and the step size *γ*, we introduced measurements from two further individual subfossil trees that were used in construction of IntCal20 [1]. A15 from the site of Avigliana in Northwestern Italy and P305u from the site of Palughetto in Northeastern Italy were measured for ^14^C on decadal blocks. They cannot be dendro-matched together and both remain floating [33]. We considered the calendar ages of A15 and P305u as unknown within our MCMC scheme, simultaneously allowing us to match these A15 and P305u single tree series to the DRM1, DRM2 and DRM3 sequences (set at their, optimal, posterior mode calendar ages). These age estimates are shown in figure 6*c* and *d*. We obtained posterior calendar age estimates for A15 suggesting a shift of 10.7 ± 2.4 (mean ± 1*σ*) cal yrs younger (posterior mode estimate of 11 cal yrs) relative to the placement published by Adolphi *et al*. [33]; and for P305u, a suggested shift of 0.7 ± 3.9 cal yrs younger (posterior mode of 3 cal yrs older). The uncertainties on these new placements constitutes a significant improvement with respect to previous (1*σ*) uncertainties of 12 cal yrs (A15) and 28 cal yrs (P305u) published by Reimer *et al*. [1] and Muscheler *et al*. [34]. These estimates were based on an analogous MCMC-based matching technique, but only using the IntCal20 data, mainly U-Th dated carbonates in this time range.

**Figure 7.**
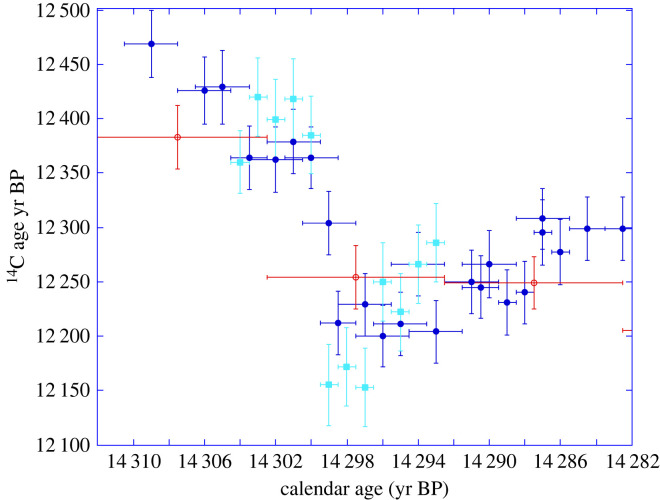
Radiocarbon ages of the two dendro-matched trees from Drouzet (DR313 dark blue circles, Drouz19 light blue squares) and the wiggle-matched tree from Italy (P305u red open dots) placed on the new chronology covering the abrupt spike, which occurs at 14 300–14 299 cal yr BP. The (*y*-axis) error bars for ^14^C ages are shown at 1–σ. The number of annual rings measured in each sample is represented by the (*x*-axis) bars (e.g. ±0.5 yr for annual ring samples for most Drouzet analyses, ±5 yr for decadal measurements).

As shown in figure 7, the abrupt event at 14.3 cal kyr BP is sampled in two Drouzet trees that are dendro-matched (DR313a and Drouz19 belonging to DRM1). DR64 covers the peak, but unfortunately this wood sampled in 2008 is not well preserved and its ^14^C cannot be analysed. Drouz19 has annual rings sufficiently large to allow ^14^C at annual resolution, while the wood of 2 or 3 years was mixed to date the tree DR313a. Both records agree in showing an abrupt drop of 200–250 ^14^C years at 14.3 cal kyr BP (equivalent to about 30‰ in Δ^14^C), followed by a gradual increase over at least a decade. In Drouz19, located at its most likely (modal) calendar age, the ^14^C event occurs between 14 300 and 14 299 cal yr BP. This abrupt change is corroborated by the few P305u data measured on decadal blocks (figures 6 and 7). The estimate for the size of the Δ^14^C step *γ* in our MCMC model, shown in figure 6*b*, allows us to assess whether this sharp change in ^14^C observed within DRM1 is simply statistical noise or a genuine atmospheric feature. The posterior for *γ* provides overwhelming evidence that the sharp jump in ^14^C observations over the course of this single year cannot solely be explained by a smooth spline—we obtain an estimate of a rise in Δ^14^C of 31 ± 4.5‰.

**Figure 8.**
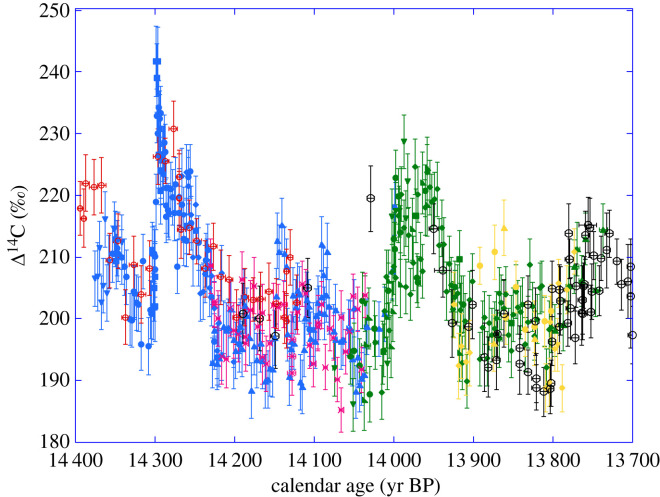
Δ^14^C records corresponding to figure 6. The (*y*-axis) error bars for Δ^14^C ages are shown at 1-σ. The data for Drouzet trees are shown for the DRM1 (blue), DRM2 (green) and DRM3 (yellow) sequences and for the P305u (red open dots) and A15 (pink crosses) Italian trees placed on the new chronology with our MCMC scheme for matching to the German pine absolutely dated chronology (black open dots). The number of annual rings measured in each sample is represented by the (*x*-axis) bars (e.g. ±0.5 yr for annual ring samples for most Drouzet analyses, ±5 yr for decadal measurements).

Figure 8 shows all data converted in terms of changes of the atmospheric Δ^14^C. As expected, the Δ^14^C is characterized by an abrupt increase at 14.3 cal kyr BP with a gradual decay and a prominent century-long maximum between 14.05 and 13.95 cal kyr BP, with amplitudes around 30‰. Figure 8 clearly shows the asymmetric shape of the 14.3 cal kyr BP spike similar in shape to the 774 CE event [15], but twice as large in amplitude.

## 6. Comparison with the ^10^Be record

A way of checking the origin of rapid ^14^C excursions is to compare these fluctuations with independent records based on other cosmogenic radionuclides such as ^10^Be in polar ice. Both cosmogenic isotopes are produced in similar ways in the upper atmosphere. However, ^14^C and ^10^Be time series cannot be compared directly since the fates of these two cosmonuclides are very different in the atmosphere: ^10^Be atoms become fixed to aerosols and are washed out by precipitation quickly after their production (within ≈ 1–3 years, [35]). ^10^Be fallout is thus a marker of the regional, latitude-dependent, cosmogenic production. By contrast, ^14^C atoms are oxidized and mixed into the atmospheric CO_2_ pool, which is connected to larger reservoirs of the carbon cycle such as the biosphere and bicarbonate ions dissolved in the oceans. This ‘dilution’ attenuates and delays ^14^C concentration variations in the different carbon reservoirs with respect to ^14^C production variations.

For the comparison with the Drouzet record, it is possible to use the relatively high-resolution ^10^Be record from the GRIP ice core from Greenland [36] which is dated by counting annual couplets [3,37]. In the time window of interest, the ^10^Be sampling resolution is decadal and the 1–σ precision of the GICC05 chronology is about 80–90 years [3,37]. Adolphi *et al*. [36] used the GICC05 age scale (in yr BP with 1950 as reference datum) and ice-flow modelling to infer changes of snow accumulation and convert the measured ^10^Be concentration in the GRIP core in terms of ^10^Be flux. As underlined by these authors, the accumulation rate changes occur mainly at climatic transitions between stadials and interstadials, implying that high-frequency changes are similar in the ^10^Be concentration and flux records.

**Figure 9.**
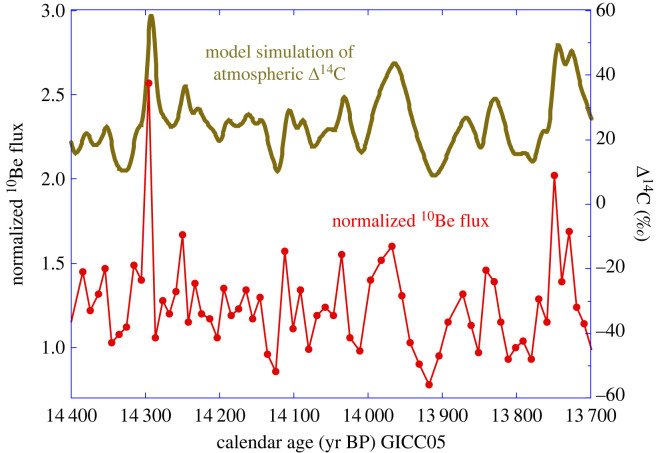
GRIP ^10^Be flux record (red dots) normalized to its average value over the 8 kyr of the Late Glacial period sampled by Adolphi *et al*. [36] who used the GICC05 chronology expressed in yr BP (with 1950 as the reference datum). The prominent maximum based on a single analysis shows a doubling of the ^10^Be flux at 14.3 kyr BP (ice bag sample between depths dated with GICC05 at 14 301 and 14 292 cal yr BP, respectively). The brown curve shows the Δ^14^C model simulation for the troposphere of our 12-box carbon cycle model [38] forced with the normalized GRIP ^10^Be flux record (red dots).

Figure 9 shows the GRIP ^10^Be flux record normalized to its average value over the 8 kyr of the Late Glacial period sampled by Adolphi *et al*. [36]. During the 14.4–13.7 cal kyr BP window the flux is rather stable, about 25% higher than the long-term average. The record shows two main multidecadal maxima between 14.0 and 13.96 kyr BP and between 13.75 and 13.7 kyr BP. The record also shows a prominent maximum based on a single analysis suggesting a doubling of the ^10^Be flux at 14.3 kyr BP (ice bag sample between depths dated with GICC05 at 14 301 and 14 292 cal yr BP, respectively). A somewhat smaller excursion is observed with the ^10^Be raw concentrations.

## 7. Data-model comparison

A quantitative comparison of ^14^C and ^10^Be requires the use of a carbon cycle model (e.g. [38–41]). The carbon cycle acts as a low pass filter because both attenuation and delay depend on the timescale of ^14^C production variations. The damping effect is such that variations of ^14^C production are attenuated by a factor of about 20 and 100 for centennial and decadal variations, respectively. The phase lag between production and atmospheric ^14^C concentration is about 12 and 2–3 years for centennial and decadal variations, respectively (e.g. fig. 3 in [41]).

Following our previous work [38,41], we have used the normalized ^10^Be record in Greenland ice as a forcing input to a 12-box carbon cycle model, whose characteristics have already been compared with other models [41,42]. This approach is similar to that followed by Adolphi *et al*. [33] who used the GRIP ^10^Be flux record of Adolphi *et al*. [36]. The calculation of ^10^Be flux has some inherent pitfalls (e.g. see specific discussion by [41]). Consequently, we used both normalized ^10^Be flux and normalized ^10^Be concentration records as input curves for our model, the preferred simulation being the one based on ^10^Be flux as in Adolphi *et al*. [33].

As a first approximation, we assumed no polar enhancement for the ^10^Be record (i.e. PEC = 1 according to [38]) and no difference in relative production modulation between ^14^C and ^10^Be. Recent work has shown that the polar enhancement due to the solar modulation is only about +8% [43]. In addition, the relative modulation of ^14^C production during solar cycles is only a few % lower than for ^10^Be according to Poluianov *et al*. [44].

**Figure 10.**
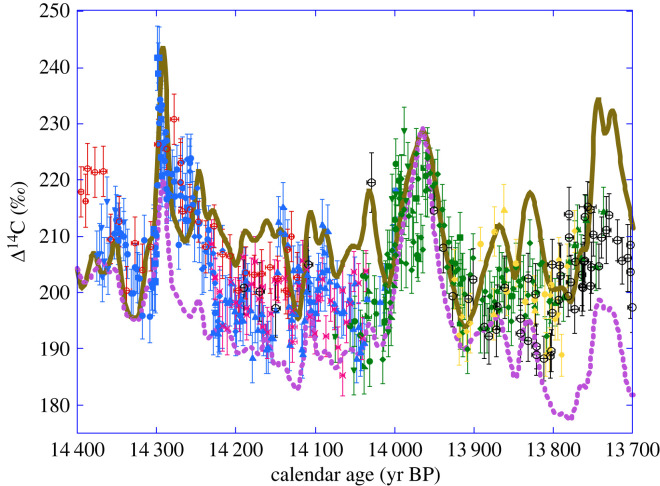
Δ^14^C records of Drouzet, Italian and German pines placed on the new chronology compared with our Δ^14^C simulations with the 12-box model forced with the normalized GRIP ^10^Be flux (brown curve) and normalized ^10^Be concentration record (dashed purple curve). Both simulations are on the GICC05 timescale and were shifted along the *y*-axis by constant Δ^14^C values to match a common Δ^14^C at 14.4 kyr BP similar to the observations. The data for Drouzet trees are shown for the DRM1 (blue), DRM2 (green) and DRM3 (yellow) sequences and for the P305u (red open dots) and A15 (pink crosses) Italian trees placed on the new chronology with our MCMC scheme for matching to the German pine absolutely dated chronology (black open dots).

Figure 9 shows the simulated Δ^14^C record we obtain from our carbon cycle model when forced by the GRIP ^10^Be flux profile. As expected, the simulated Δ^14^C has a much lower amplitude than the ^10^Be record, as well as being smoother and slightly delayed. The main features in the simulated Δ^14^C are two, century-long, maxima between 14 and 13.9 kyr BP and 13.75 and 13.65 kyr BP and a sharp maximum at 14.3 kyr BP with an amplitude on the order of 30‰. Figure 10 shows our (^10^Be-based) simulations of Δ^14^C compared with the observed Drouzet Δ^14^C record. Both simulations were shifted along the *y*-axis by constant values to match a Δ^14^C value at 14.4 kyr BP similar to the data. The agreement between model and data is satisfactory for the three main features, although small differences exist in amplitude and phase. The latter may be due to small discrepancies between the independent ice core and tree ring chronologies.

## 8. The 14.3 cal kyr BP event as a new SEP spike

**Figure 11.**
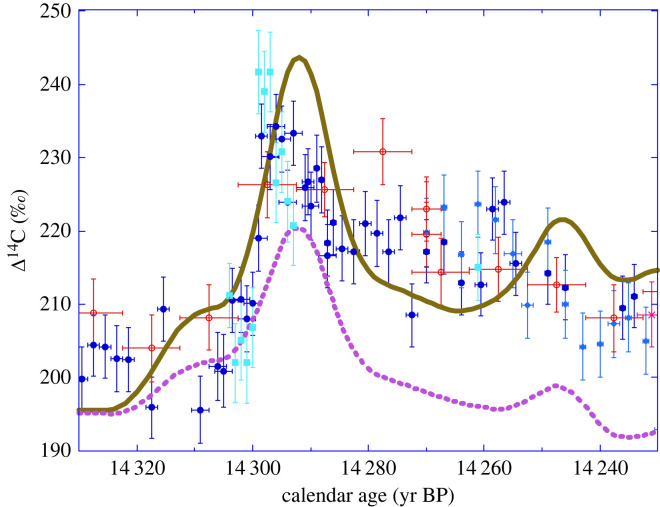
The same as figure 10 but focused on the 14.3 cal kyr abrupt event. Δ^14^C records of Drouzet, Italian and German pines placed on the new chronology compared with our Δ^14^C simulations with the 12-box model forced with the normalized GRIP ^10^Be flux (brown curve) and normalized ^10^Be concentration record (dashed purple curve). Both simulations are on the GICC05 timescale and were shifted along the *y*-axis by constant Δ^14^C values to match a common Δ^14^C at 14.4 kyr BP similar to the observations. The data for Drouzet trees are shown for the DRM1 (blue) sequence, notably DR313 in dark blue and Drouz19 in light blue, and for the P305u (red open dots) and A15 (pink cross) Italian trees placed on the new chronology.

A more detailed view is provided in figure 11 to study the 14.3 kyr BP event. There is a general agreement in timing and amplitude between the simulated and observed Δ^14^C, although the period around 14.27 kyr BP is characterized by a 10‰ residual difference.

The data-model comparison confirms the abrupt 30‰ increase linked to cosmogenic production. The simulated (^10^Be-based) maximum in Δ^14^C occurs at 14 292 cal yr BP, i.e. 7 cal yrs later than in the observed Δ^14^C record. This difference is much smaller than the 85 cal yrs uncertainty at 1-σ of the GICC05 ice core chronology. Moreover, 7 cal yrs is even smaller than the time span of 9 cal yrs represented by the ice bag from the GRIP core analysed for its ^10^Be content that shows an elevated value [36]. It is obvious that shifting the GICC05 chronology by 7 cal yrs towards older ages would result in a perfect synchrony between data and model. We did not apply such an ad hoc correction because it is somewhat pointless given the decadal resolution of the ^10^Be record.

Another issue is that the simulated Δ^14^C wiggle is symmetric, unlike the observed spike with its sharp rise and gradual relaxation. This difference may also be due to the decadal resolution of the ^10^Be record, implying that its spike is represented by a triangular increase over 20 years (twice the sampling step). Assuming an equivalent ^10^Be flux anomaly over a single year would result in a sharp Δ^14^C increase in a year, of larger magnitude followed by a gradual decrease.

An additional difficulty in the simulation is the lack of precise knowledge on the cosmogenic production during a SEP event. The common ^14^C production by galactic cosmic rays occurs mainly in the lower stratosphere (about two-thirds or less) and in the upper troposphere (about one-third or more) with a latitude dependence linked to the geomagnetic cut-off rigidities. According to Golubenko *et al*. [45], the ratio between stratospheric and tropospheric productions and the latitudinal dependence may have been different for SEP events, during which production would have occurred mainly in the high-latitude stratosphere.

Another limitation of the data-model comparison is that ^10^Be variations in polar ice cores may under-represent the cosmogenic production variations linked to geomagnetic field changes. The relative geomagnetic modulation of cosmogenic production is greatest at the Equator, and lowest at the poles [44–46,48]. Although most ^10^Be production occurs in the stratosphere characterized by intense horizontal mixing and a relatively long residence time [49–51], any deviation from complete homogeneous atmospheric mixing would affect the relative amplitudes of the geomagnetic and solar signals embedded in ^10^Be records. Recently, Adolphi *et al*. [43] evaluated that geomagnetic variations are dampened by 23–37% in polar ice records. Such a polar atmospheric dampening of the geomagnetic modulation would not occur for ^14^C, which is mixed globally.

Unfortunately, there is a lack of precise and high-resolution paleomagnetic intensity records for the 14.4–13.7 kyr BP window, and available records strongly disagree over the last deglaciation (see [52] for a review). In any case, the intensity of the geomagnetic field usually varies slowly on timescales longer than centuries, implying that they could not have contributed to the decadal fluctuations of ^14^C and ^10^Be observed between 14.4 and 13.7 cal kyr BP, notably the abrupt 14.3 cal kyr BP spike. Furthermore, Fournier *et al*. [53] ruled out an imprint on geomagnetic production by short-term geomagnetic jerks, whose existence and global significance are still debated.

Given the limitations of the 14.3 cal kyr BP event in the GRIP ^10^Be record and the multiple uncertainties listed above, it seems premature to make further tests to improve the fit between observed and simulated data for this abrupt event. Nevertheless, the fact that it occurs in a single year in the Δ^14^C record (14 300–14 299 cal yr BP) concomitant with a ^10^Be anomaly makes it a probable SEP event with the largest magnitude evidenced so far, twice that of the eponymous 774 CE event [15].

## 9. The 14 cal kyr BP century-long ^14^C and ^10^Be excursion and the Older Dryas climate event

**Figure 12.**
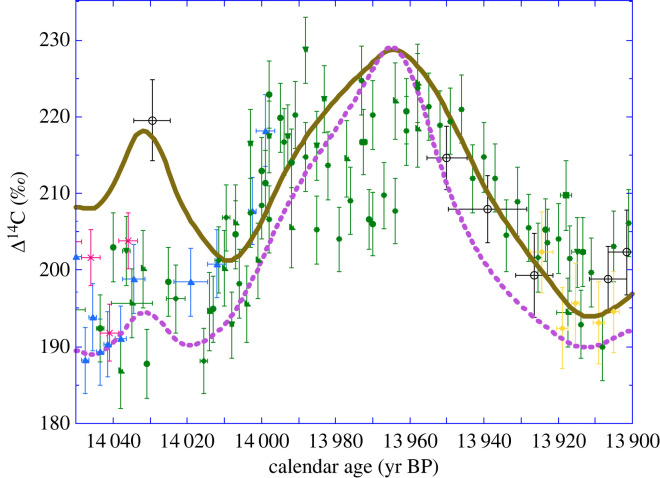
Same as figure 10 but focused on the 14 cal kyr event. Δ^14^C records of Drouzet, Italian and German pines placed on the new chronology compared with our Δ^14^C simulations with the 12-box model forced with the normalized GRIP ^10^Be flux (brown curve) and normalized ^10^Be concentration record (dashed purple curve). Both simulations are on the GICC05 timescale and were shifted along the *y*-axis by constant Δ^14^C values to match a common Δ^14^C at 14.4 kyrBP similar to the observations. Colour codes, labels and errors are the same as in previous figures. The data for Drouzet trees are shown for the DRM1 (blue), DRM2 (green) and DRM3 (yellow) sequences, for the A15 (pink crosses) Italian trees placed on the new chronology with our MCMC scheme for matching to the German pine absolutely dated chronology (black open dots).

Figure 12 illustrates the agreement between our observed Drouzet and the (^10^Be-based) simulated Δ^14^C during an excursion between 14 010 and 13 910 cal yr BP. The correspondence in amplitude (30‰) strongly suggests that the excursion is linked to cosmogenic overproduction over the course of a century, similar to the frequent Maunder-type solar minima of the last millennia (e.g. [13,14,38]).

The interesting aspect of this 14 kyr solar minimum is that it corresponds precisely with the brief climatic event called the Older Dryas, which separates the longer Bølling and Allerød mild phases [54]. The literature on this specific climatic event is limited as most geological archives are unlikely to provide the resolution, dating control and spatial representativity to study such a century-long event. Nevertheless, the Greenland ice core records clearly show a brief cooling from 14 025 to 13 904 years BP (with a 1–σ uncertainty of 85 years) based on the ∂^18^O measured in ice and on other chemical proxies (e.g. [55,56]). By combining proxies, notably nitrogen isotopes (∂^15^N) of entrapped air, which are sensitive to local temperature and temperature gradient in the ice firn, Kindler *et al*. [57] estimated a cooling of *ca* 6–7°C during the Older Dryas, about half to two-thirds of the cooling experienced during the longer cold phases called the Oldest Dryas (≈ Heinrich 1 stadial) and the Younger Dryas.

The causes of these two, millennium-long, events are the subject of a large amount of literature mainly dealing with the impact of glacial meltwater from the North American and North European ice sheets, which disappeared during the last deglaciation. By contrast, the exact causes of the Older Dryas are largely unknown and unexplored.

The 14 cal kyr BP solar minimum could have contributed to the cooling but the overall amplitude of the total solar irradiance change expected from such a Maunder-type solar minimum is clearly insufficient [14,41,58–60]. Direct solar forcing cannot be the sole explanation, but it should be noted that the climate system may have been more sensitive during the Late Glacial period. Indeed, by using a coupled climate system model, Braun *et al*. [61] simulated a strong response to small periodic solar changes due to strong nonlinear dynamics combined with the global Meridional Ocean Circulation (MOC).

An alternative explanation could be that ^14^C and ^10^Be anomalies observed during the Older Dryas are just consequences of the climatic change and not solar proxies that can be used to identify its cause. The Older Dryas is characterized by a specific drop of ice accumulation [62], which may have biased the estimation of ^10^Be flux changes. Nevertheless, the 14 kyr BP anomaly is indeed present in both the ^10^Be concentration and flux records [36] and the simulation based on ^10^Be flux agrees with that based on ^10^Be concentrations (figure 12), suggesting that the ^10^Be excursion at 14 kyr BP is genuine and not an artefact of ice accumulation rate changes.

Both the Younger Dryas and Oldest Dryas are characterized by collapse of the MOC. If this was also the case during the Older Dryas, this could have led to an increase of the atmospheric Δ^14^C. Steady-state calculations based on the 12-box model [38] indicate that halving the MOC strength from 20 to 10 Sv (1 Sv = 10^6^ m^3^ s^−1^) leads to a Δ^14^C increase of 35‰, in broad agreement with observations for the 14 kyr BP event. A similar calculation can be performed with a box-diffusion model [63]: decreasing the oceanic eddy diffusivity from 4000 to 3000 m^2^ yr^−1^ leads to an increase of the atmospheric Δ^14^C by 40‰ accompanied by a 320 yr increase of the mean ^14^C age of the deep ocean, about half of what is observed for the last glacial maximum [64]. However, these are steady-state calculations, and the atmospheric pool does not settle instantaneously to its steady-state Δ^14^C value. Indeed, all reservoirs exchange with each other and the atmosphere rises slowly over centuries (e.g. [65,66]). Using the 12-box model to simulate a MOC drop by a factor of two over a century leads to a modest and gradual rise of 8‰ in Δ^14^C during the event followed by a slow decrease during the centuries after the event.

Although the timing and amplitude of MOC changes during the Older Dryas event are hypothetic and speculative, they fall short in explaining the amplitude and shape of the observed ^14^C and ^10^Be excursions at 14 cal kyr BP, the most parsimonious explanation being a common Maunder-type solar minimum.

## 10. Conclusion and perspectives

Subfossil pines found in the banks of the Drouzet watercourse in the region of the middle course of the Durance River in the Southern French Alps allowed us to construct a ≈ 700-yr long floating chronology dated by ^14^C mostly on individual annual rings.

Matching of the new Drouzet record with the German pine absolutely dated chronology provides an accurate placement of the Drouzet record between 14.4 and 13.7 cal kyr BP. The length of the Drouzet record, and its long overlap with the German pine chronology, allows us to calculate a 1-σ uncertainty of *ca* 2.5 cal yrs for the Drouzet chronology. The Drouzet record allows to extend the radiocarbon calibration on tree rings by *ca* 500 years beyond its present limit of 13 900 cal yr BP.

The resulting atmospheric Δ^14^C is characterized by an abrupt increase at 14.3 cal kyr BP with a gradual decay and a prominent century-long maximum between 14.05 and 13.95 cal kyr BP, both with amplitudes around 30‰.

The 14.3 cal kyr BP event starts abruptly with a sharp Δ^14^C rise in a single year, which makes it a probable SEP event with the largest magnitude of all those SEPs identified so far. Critically, the high calendar age precision we obtain from our matching to the German pines propagates through to the date of the spike. Using our MCMC approach to matching, we estimate that the SEP spike was most likely to have occurred in the calendar year between 14 300–14 299 cal yr BP (with a 68% or 1–σ, probability that it occurred in one of the years between 14 301 and 14 296 cal yr BP).

The SEP spike evidenced in the Drouzet record corresponds to a ^10^Be anomaly in Greenland ice dated between 14 301 and 14 292 cal yr BP [36], with a 1–σ chronological uncertainty of 85 yrs [3].

The Drouzet Δ^14^C record also shows a century-long maximum between 14 010 and 13 910 cal yr BP, which is synchronous with a similar event in the simulated Δ^14^C record based on the Greenland ^10^Be record. The correspondence in amplitude (30 ‰) suggests that the excursion is linked to a cosmogenic overproduction during a Maunder-type solar minimum.

The comparison of the two prominent events in both ice and tree records at 14.3 and 14 cal kyr shows that the time difference between the independent Greenland ice and tree-ring chronologies is only a decade, at the most, in this time range. Such a close agreement may be fortuitous given the large calendar uncertainty in ice cores, but it is reassuring for the identification and interpretation of common causes in both cosmogenic isotope records.

Further progress in identifying causes will come from replicating the ^14^C record in other subfossil trees from other locations, notably in the Southern Hemisphere. In addition, our study underlines the need to increase the time resolution of sampling in polar ice cores in order to go towards the annual resolution of the tree-ring record. Ice core records from Antarctica will also be useful to constrain the variations of ^10^Be flux and to reconstruct cosmogenic production changes.

## Data Availability

Additional data are available as electronic supplementary material in tables S1, S2 and S3 [67].
